# Reactive case detection of *Plasmodium falciparum* in western Kenya highlands: effective in identifying additional cases, yet limited effect on transmission

**DOI:** 10.1186/s12936-018-2260-2

**Published:** 2018-03-13

**Authors:** Ebenezer K. Aidoo, Yaw A. Afrane, Maxwell Gesuge Machani, Winnie Chebore, Bernard Walter Lawson, Harrysone Atieli, Simon Kariuki, Ming-Chieh Lee, Cristian Koepfli, Guofa Zhou, Andrew K. Githeko, Guiyun Yan

**Affiliations:** 1 Department of Theoretical & Applied Biology, College of Science, Kwame Nkrumah University of Science & Technology, Kumasi, Ghana; 2 Kenya Medical Research Institute/Centre for Global Health Research, Kisumu, Kenya; 3 Department of Medical Microbiology, College of Health Sciences, University of Ghana, Korle-Bu, Accra, Ghana; 4 School of Public Health, Maseno University, Maseno, Kenya; 5 Program in Public Health, College of Health Sciences, University of California, Irvine, CA 92697 USA

**Keywords:** Reactive case detection, Index case, Asymptomatic parasite carriers, Neighbourhood

## Abstract

**Background:**

Identifying asymptomatic reservoirs of malaria parasites using index cases as entry points into the community is potentially a cost-effective way towards achieving malaria elimination.

**Methods:**

Within 1 year, 1430 confirmed malaria cases were identified in Marani hospital, western Kenya. Fifty cases were followed up, and 108 index case household members and 612 neighbours within a 100 m radius were screened. As controls, samples were collected from 510 individuals matched with index cases and located at a distance of ≥ 500 m from them. Infections were diagnosed by microscopy and PCR while simultaneously collecting malaria vectors indoor using pyrethrum spray catches.

**Results:**

In the index case and neighbour households, the prevalence of infection was approximately twice as high as in control households (by PCR: index cases households: 28.9%, neighbours: 25.3%, matched controls: 12.9%). In index case households, the indoor vector density (*Anopheles gambiae* and *Anopheles funestus*) was higher (0.46 female/house/night) than in neighbouring (0.31 f/h/n) and control houses (0.29 f/h/n).

**Conclusions:**

Screening index case households and neighbours approximately doubles the chance to detect asymptomatic infections compared to randomly selected households. However, even if all cases were followed up, only a small proportion (˂ 10%) of the asymptomatic reservoir in the population would have been identified. Control programmes need to weigh the increased chance to find cases around index cases vs. the logistical challenges to target this subgroup within the population.

## Background

Given the efforts made in the past decade in the fight against malaria, the spotlight on malaria intervention strategies has changed from malaria control to elimination in many regions of the world [1, 2]. This is also the case in the highland areas in western Kenya. In the past decade, the Roll Back Malaria Partnership through its mass distribution campaigns was able to achieve long-lasting insecticide net (LLIN) coverage of ≥ 80% in western Kenya, though usage might remain lower [3]. The intensive malaria control campaign has led to a significant decline in malaria prevalence in this hypoendemic area [4]. However, the most recent study showed an upsurge of malaria in Western Kenya [5] and asymptomatic infection is common and can sustain transmission [6–8]. These asymptomatic infections are not targeted by control programmes focusing on passive case detection in health facilities.

Infections are often spatially clustered in households or homesteads, which might be at increased risk of transmission because of proximity to vector breeding sites, or because of occupation and behaviour of their residents [9–11]. In this context, new control measures may be needed to further reduce malaria transmission [12]. Active case detection (ACD) strategies for malaria elimination has been recommended by the World Health Organization [12]. Reactive case detection (RACD) aims to screen individuals living near clinical cases (index cases) diagnosed at health facilities, as they represent foci of infections [13, 14]. This approach has shown effectiveness in detecting extra infections because of spatial clustering of infections within houses and neighbours [15–18]. For example, in Belize, 50% of malaria cases occurred in only 8% of households [19]. Hence, infections are observed at higher prevalence in households in the neighbourhood of index cases relative to those further away [20]. This offers the possibility to achieve a substantial reduction in transmission by conducting activities such as IRS or mass drug administration in a small number of households. In the case that infections are acquired predominantly outside of the home, however, RACD has shown limited effectiveness [11].

Reactive case detection presents a number of logistical challenges. The household location of each clinical case needs to be recorded and communicated to teams ready to conduct follow-up activities. The identification of the size of foci, and thus the number of households to target, requires a detailed understanding of the transmission epidemiology. The present study focused on reactive case to detect asymptomatic infections—i.e. infections that did not result in treatment seeking of the carrier—of malaria in the Western Kenya highlands, and compared the number of secondary infections detected in index case households and neighbours to the estimated total of infections in the community.

## Methods

### Study site

**Fig. 1:**
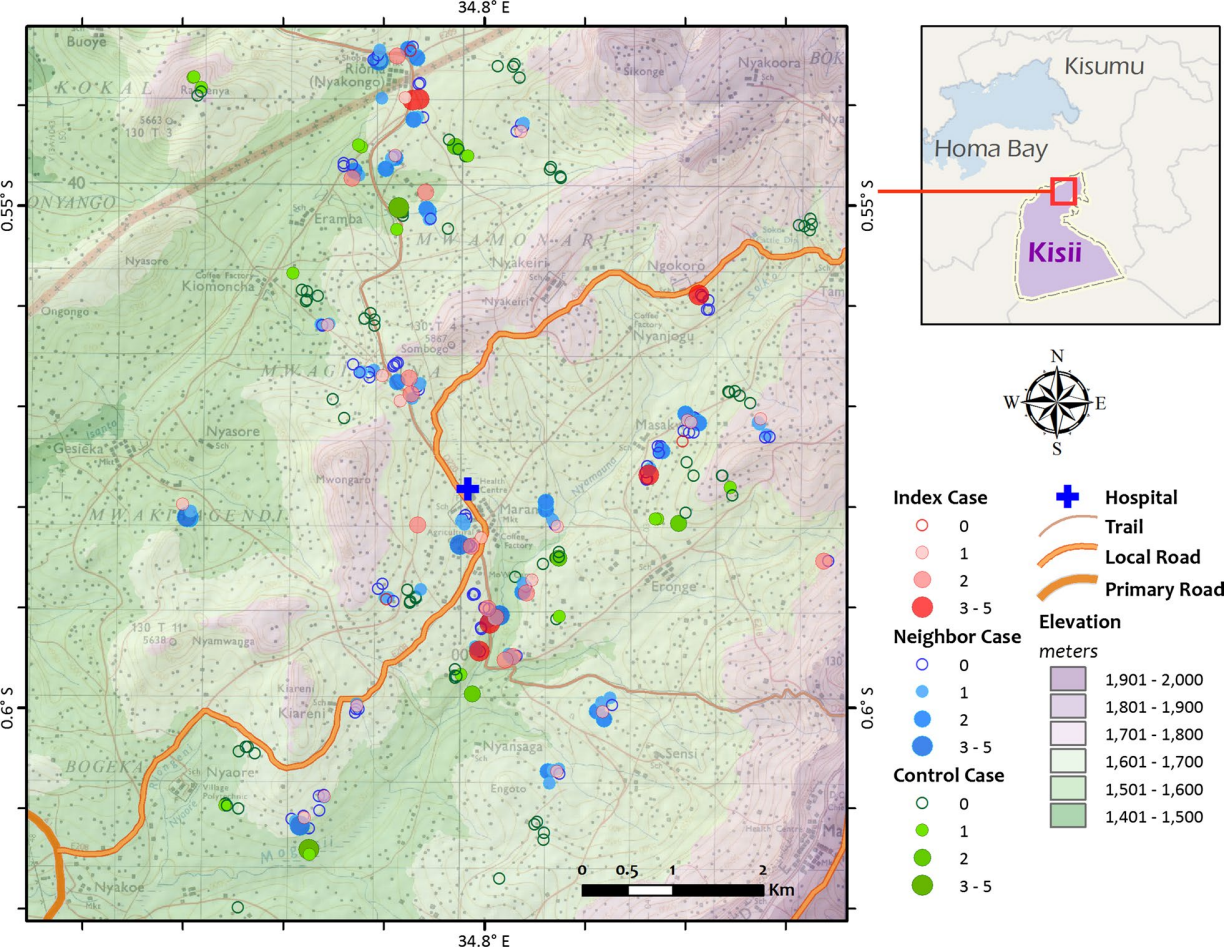
Study site. Map of study site with case households (red), neighbouring households (blue), and control households (green). The size of the circle represents the number of infections (by qPCR); households without infection are represented by empty circles

The study was conducted in Marani, Kisii County, Western Kenya highlands, between October 2015 and August 2016 (Fig. 1). Marani Hospital (34°48′9″E, 00°35′9″S, and 1540–1740 m above sea level), the only sub-county hospital in the area, was used as the recruiting centre for the index cases. The catchment population of the hospital is about 100,000 individuals. In addition, the catchment area is served by four health posts. It is estimated that half of all malaria cases present to the health posts and Marani Hospital.

The area has pockets of forests by the side of the rivers and streams, which are remains of a larger forest that has been deforested for farming and grazing. The valley is marked by an effective drainage system and floods are unusual. The type of housing and roofing includes grass-thatched, mud, wood, walls made of brick or stone and iron sheet roofs. Marani is under low and unstable malaria transmission and thus described as hypoendemic for malaria [4]. The climate in western Kenya typically comprises a bimodal pattern of rainfall, with the long rainy season from April to June triggering the peak malaria transmission period. The short rainy season is from October through November. The dry season is from July to September with January and February as the driest and hottest months. Mean yearly rainfall and temperature ranges from 1800 to 2000 mm and 17–20 °C respectively [21]. *Plasmodium falciparum* is the main malaria parasite species [22], with the major malaria vector species as *Anopheles gambiae* s.s. and *Anopheles funestus* [23].

### Study design

Between October 2015 and September 2016, all confirmed malaria cases in Marani hospital were recorded, and 50 microscopically confirmed cases were followed up to their residence for active case investigation. All members of the index case household, and individuals living within a 100 m radius (five nearest neighbouring households) were screened.

To estimate the prevalence of asymptomatic malaria in the population, for each index case, control individuals from 5 households ≥ 500 m of the index case were screened (after confirming that no one in the household had symptoms of malaria, and absence of confirmed malaria in the past 11 months). In addition, samples were available from school children in the Marani sub county. 92 samples each collected in September 2015, April 2016, and July 2016 were screened by qPCR.

Case investigation and RACD was initiated within 7 days of the index case detection. Except for 1 month (June) of no sample collection by the research team, participants were screened for 6 days every month with a screening rate average of 25 per day (inclusive of matched controls). Consented members of the index case household, neighbouring and control households available at the time of the survey were screened. Those members of these households not present during the initial visit were followed up for a maximum of three visits (1 per month) to ensure maximum representation. Simultaneously, indoor resting mosquitoes were collected in all the study households using pyrethrum spray catches [24]. Whenever possible, pyrethrum spray catches were undertaken in the morning, prior to cooking in these households.

### Data collection

Hand held global positioning system (eTrex, Vista, Garmin, USA) receivers were used to take elevation and location data of sites visited. Information collected on the structured administered questionnaire included age, gender, bed net ownership, whether the household has been sprayed with insecticide, drug use, recent fever and travel history. Irrespective of recent fever history, blood specimen was collected by a single finger prick, spotted on Whatman 903™ protein saver card for PCR analysis and smears prepared for thick and thin blood films.

Using the keys of Gillies and De Mellion [25], collected *Anopheles* were identified and separated into species, sex and counted. Females were further subdivided as unfed, blood fed, half-gravid and gravid based on the condition of their abdomen as described by Detinova [26].

### Laboratory methods

Thick and thin blood films were stained with 10% Giemsa, followed by determination of malaria parasitaemia status and density. Negative results were based on examination of 100 high power fields. For every positive thick blood film, parasitaemia level was estimated by counting at least 200 white blood cells and assuming a white blood cells count of 8000 per microlitre [27, 28]. Quality control was achieved by staining a known positive and negative sample to ascertain the quality of Giemsa for each freshly prepared stock [28]. Dried blood spots on Whatman 903™ protein saver card were inserted into individual sealed zip locks containing desiccant and stored at − 20 °C for qPCR. DNA was extracted using the Chelex method as previously described [29, 30] and stored at − 20 °C. *P. falciparum* qPCR was done according to published protocols [31]. CS-ELISA method was used to detect sporozoites [32].

### Statistical analyses

Questionnaire, parasite and entomological data were entered into Microsoft excel with demographics and infection rates of the index case compared with the other groups. χ^2^ test was used to determine prevalence differences in age groups. The indoor resting density of mosquito species was calculated as number of females per house per night (per survey) [23]. Difference in mean vector density between study groups was compared using generalized linear model (GLZ) assuming negative binomial distribution. Since samples were likely locally clustered for each index case (rather than randomized) due to the nature of sampling design, all samples for each index case were treated as a cluster, a covariate, in the GLZ model.

### Ethics

The study was approved by the Kenya Medical Research Institute Scientific & Ethics Review Unit and the Institutional Review Board at the University of California, Irvine. Informed consent was sought from all adult individuals and assent form administered to individuals below 18 years and signed by their parents or guardians to consent. All individuals who tested positive for malaria during household visits were referred to the Marani Hospital and treated according to current national malaria treatment guidelines.

## Results

### Number of clinical cases at Marani Hospital

**Fig. 2:**
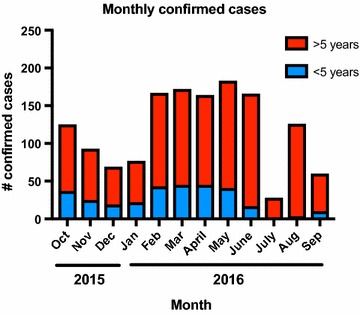
Monthly confirmed cases

Within a period of 12 months between October 2015 and September 2016, a total of 5138 clinical malaria cases presented to Marani hospital, of which 1430 (27.8%) were confirmed by microscopy or RDT. There was moderate seasonal variation of cases, with the highest number of confirmed cases between February and June (Fig. 2). In most months, approximately 0.2% of the population presented with febrile illness to the hospital and malaria infection was confirmed.

### Infections identified by reactive case detection

In the frame of the RACD activities, 1280 individuals residing in 413 households were screened: 50 passively detected index cases, 108 individuals from index case households, 612 individuals from neighbouring households, and 510 from control household, with a mean of 3.7, 3.0, 3.0 participants screened per household. Demographic and clinical details of the study groups are given in Tables 1 and 2.

**Table 1:** Characteristics of the study population

Parameters	Study group			*P* value^§^	
	Index case household	Neighbour	Control	I vs. N	I vs. C
Total homesteads screened	43	202	168		
Total population screened	158	612	510		
Median age (years)^a^	11	15	15	0.802	0.802
Parasite prevalence (% individual)					
PCR	28.9	25.3	12.9	0.484	< 0.001
Microscopy	8.3	10.3	4.7	0.708	0.059
Age group (% individual)					
< 4.9	29 (18.4)	124 (20.3)	77 (15.1)		
5 ~ 14.9	62 (39.2)	170 (27.8)	165 (32.4)	0.018	0.084
≥ 15	67 (42.4)	318 (52.0)	268 (52.5)		
Sex (% individual)^b^					
Male	63 (39.9)	230 (37.8)	205 (40.5)	0.624	0.888
Female	95 (60.1)	379 (62.2)	301 (59.5)		
Occupation (% individual for age ≥ 15 only)					
Sample size^c^	67	318	268		
Peasant farmer	49 (73.1)	227 (71.4)	176 (65.7)		
Employed	3 (4.5)	16 (5.0)	20 (7.4)		
Student	9 (13.4)	38 (11.9)	35 (13.1)	0.865	0.562
Other	0 (0.0)	5 (1.6)	6 (2.2)		
Unknown	6 (9.0)	32 (10.1)	31 (11.6)		
Bed net ownership (% household)	137 (86.7)	554 (90.1)	477 (93.5)	0.159	0.006
Use of bed net (% individual)	128 (81.0)	510 (83.3)	439 (86.1)	0.488	0.121
Travel within previous 14 days	0 (0.0)	3 (0.5)	7 (1.4)	0.501	0.149

^§^P value: column I vs. N—comparison of index case household vs. neighbour and column I vs. C—comparison of index case household vs. control

^a^Data analyzed using Kruskal–Walls H test

^b^Records missing: 3 from neighbouring and 4 from control groups

^c^For age ≥ 15 only

**Table 2:** Reported fever and use of antimalarial

Parameters	Index case	Index case household	Neighbour	Control
Reported taking antimalarial (% individual) ^†^	41 (82.0%) a	82 (75.9%) a	66 (10.8%) b	2 (0.4%) c
Reported fever (% individual)^†^	46 (92.0%) a	82 (75.9%) b	69 (11.3%) c	0 (0.0%) d

^†^Columns in the same row that are not connected by the same letter represent significantly different from each other at significant level of 0.05

By microscopy, of the 50 index cases, 49 had *P. falciparum* infection with 1 mixed infection of *P. falciparum* and *Plasmodium malariae*. qPCR for *P. falciparum* was done on 48 index cases (no DNA was available from 2 samples) and confirmed 41/48 (85%) cases.

In the index case households, RACD by microscopy identified 9 secondary cases out of the 108 individuals screened (8.3%) (*P. falciparum*: 7/108, *P. malariae*: 1/108*, P. falciparum* and *P. malariae* mixed: 1/108). In the neighbouring households, 63/612 (10.3%) individuals were positive (*P. falciparum*: 54/612, *P. malariae*: 5/612, *P. falciparum*/*Plasmodium ovale* mixed: 1/612, *P. falciparum*/*P. malariae* mixed: 3/612). In the control households, 24/510 (4.7%) of those screened by microscopy were positive. By microscopy, a gametocyte prevalence of 4% and 0.8% was found in the index cases and across the study population, respectively.

By *P. falciparum* qPCR, 24 infections in 93 (25.8%) index case household members were detected, 120 in 478 (25.1%) neighbouring households, and 34 in 263 (12.9%) control household members. Differences in prevalence of infection between age groups were moderate (Table 3).

**Table 3:** Comparison of malaria infection prevalence by qPCR (%) between age groups and study groups

Age	Index household	Neighbour	Control	Index household	Neighbour	Control
Malaria parasite prevalence				Odds ratio and 95 CI		
0–4.9	25.0	22.1	14.6	1.94 [0.48, 8.08]	1.66 [0.62, 4.42]	1.0
5–14.9	32.0	32.6	12.8	3.22 [1.14, 9.06]*	3.30 [1.62, 6.70]**	1.0
≥ 15	28.6	22.8	12.5	2.80 [1.20, 6.55]*	2.06 [1.13, 3.77]*	1.0
Odds ratio and 95 CI						
0–4.9	1.0	1.0	1.0			
5–14.9	1.41 [0.34, 5.78]	1.70 [0.94, 3.07]	0.85 [0.30, 2.46]			
≥ 15	1.20 [0.32, 4.27]	1.04 [0.60, 1.80]	0.83 [0.30, 2.29]			

Significance level: * P < 0.05, ** P < 0.01

### Proportion of all infections in the community detected through RACD

The number of asymptomatic infections that could potentially be identified by RACD was compared to the total number of asymptomatic infections in the community. Prevalence (by PCR) of asymptomatic infection in individuals living ≥ 500 m from index cases was 12.9%. Thus, in the 100,000 individuals in the catchment area, an estimated total of 12,900 infections were present at any time.

Reactive case detection identified a total of 144 additional cases in index case and neighbour households, when 50 cases were followed up, i.e. approximately 3 per index case. To identify these cases, a mean of 14.4 individuals were screened per index case. In most months, between 70 and 160 cases were identified at Marani hospital. If all of them were followed up, and three additional cases had been identified, approximately 200–500 secondary cases (assuming three secondary cases per index case) would have been identified each month through screening of 1000–2400 individuals (assuming 14.4 people screened per index case). Compared to the estimated total of 12,900 infections in the catchment population of the hospital, the 200–500 secondary cases would represent 1.5–3.9% of all asymptomatic infections.

### Vector species abundance and indoor resting Anopheline densities

Vectors were trapped in 413 houses across the study population during one night per household. Pooled vector density of 0.46, 0.31 and 0.29 females per house per night in the index case household, neighbouring and control households was recorded (Table 4) as per WHO [33]. Results of generalized linear model (GLZ) analysis indicated no difference in vector species or total vectors between study groups (Table 4).

**Table 4:** Vector density (female/house/night)

Study group	Mean density (95% CI)		
	*An. gambiae*	*An. funestus*	Total
Index HH	0.09 [0.00, 0.24]	0.37 [0.15, 0.60]	0.46 [0.17, 0.76]
Neighbouring HH	0.13 [0.06, 0.20]	0.18 [0.08, 0.29]	0.31 [0.18, 0.45]
Control HH	0.11 [0.04, 0.19]	0.18 [0.06, 0.29]	0.29 [0.14, 0.44]

## Discussion

In the present study, either by microscopy or PCR approximately twice as many asymptomatic infections were detected in index case households and in households in close vicinity, as compared to control households. These results are in line with reports from Zambia [15], Swaziland [16], Brazil [17], and the Thai-Myanmar border [18]. While the difference in infection prevalence between index case households and controls differed among studies, it was always higher in index case households.

Nevertheless, the results show that only a small proportion of all asymptomatic infections in the population could be captured by RACD. Extrapolating the numbers of asymptomatic infections in control households around index cases (12.9%) to the 100,000 individuals in the catchment area of the hospital indicates that approximately 13,000 individuals were carrying asymptomatic infections. If all confirmed cases from Marani hospital were followed up during a full month, less than 5% of infections in the population could be identified. It is estimated that at the hospital approximately 50% of cases were identified, with the remaining 50% at the four health posts serving the area.

Even when all cases presenting to health post were to be followed up, the projected number of secondary cases would rarely exceed 10% of all infections. A detailed understanding of the duration of infections, the total number of individuals infected over time, and on temporal variation of foci of transmission would be required to estimate the total number of infections detected over an extended period of time, e.g. over a full year. It also remains to be shown whether RACD could identify a larger proportion of all infections if prevalence was much lower. Studies done in regions of lower transmission have yielded mixed results, and in some cases even found higher prevalence in control households than in and around index case household [34, 35]. In the present study only moderate differences of infection prevalence between age groups were observed, thus individuals of all ages should be included in RACD activities.

Control programmes need to carefully evaluate whether RACD strategies are more efficient than population-wide control activities. Up to three follow-up visits were required to capture most individuals in index case and control households. In addition, the number of index cases fluctuates considerably over time, thus preparing the right number of teams for follow-up activities can be challenging.

On the other hand, higher prevalence of infection around index cases shows that clinical cases indeed represent foci of transmission. By microscopy only very few gametocytes carriers were identified, but mosquito feeding studies have shown that individuals that are positive by microscopy for asexual stages only are often able to infect mosquitoes, and eventually even complete submicroscopic infections [36, 37]. Thus, many of the infections identified by RACD are expected to contribute to transmission. Targeting the foci identified by RACD by spraying of insecticides or larvicides might prevent onward transmission of these cases and might be more effective than screening for secondary cases.

The current study showed clearly that prevalence within a radius of 100 m around index case households remained similarly high as in the index case households. Further studies will be needed to determine the size of foci. In parallel to the present study, school children residing in Marani were screened by qPCR (Zhou et al. unpublished). Notably, prevalence of infection was only 6.9%, i.e. approximately half the prevalence of infection in individuals living 500–1000 m from index cases and screened in parallel with them. The lower prevalence is particularly surprising, as children were often found to be at higher risk of infection [38]. This might indicate that these controls still lived in areas of higher transmission, and that foci of transmission might span across several kilometers. Similar findings were made in other sites in Kenya [10, 39].

Approximately thrice as many index case than control households reported not using a bed net, and—though the difference did not reach significance—more mosquitoes were caught in index case households. This indicates that a small number of households not using bed nets might contribute substantially to residual transmission, and corroborates the fact that control programmes should aim for 100% bed net coverage. However, a substantial proportion of *An. funestus*, the major vector in this study, might be biting and resting outdoors [23, 40] and maintain transmission [41]. Tools to control vectors outdoor will thus be crucial.

## Conclusion

Following-up clinical index cases resulted in the identification of foci of transmission with considerably higher prevalence of asymptomatic infection than the general population. Comparing the number of secondary cases identified to the overall population prevalence, however, showed that very small proportion of all infections in the population could be identified by RACD. Given the logistical challenges to achieve high coverage of RACD, control programmes need to weigh the increased chance to detect secondary cases vs. activities targeting the whole community, which might be more cost effective.
